# Inhibition of proliferation and migration of luminal and claudin-low breast cancer cells by PDGFR inhibitors

**DOI:** 10.1186/s12935-014-0089-5

**Published:** 2014-09-05

**Authors:** Leanne Stalker, James Pemberton, Roger A Moorehead

**Affiliations:** Department of Biomedical Sciences, Ontario Veterinary College, University of Guelph, Guelph, ON N1G2W1 Canada; Department of Biomedical Sciences, Ontario Veterinary College, University of Guelph, 50 Stone Road East, Guelph, ON N5A7Z1 Canada

## Abstract

**Background:**

Platelet-derived growth factors (PDGFs) bind to two receptors, PDGFRα and PDGFRβ to mediate cell proliferation, migration and survival. Although epithelial cells typically do not express high levels of PDGFRs, their expression has been reported to increase in breast cancer cells that have undergone epithelial to mesenchymal transition.

**Methods:**

PDGFR signaling was inhibited using Sunitinib malate, Imatinib mesylate or Regorafenib in murine and human luminal-like and claudin-low mammary tumor cell lines or Masitinib in only the human cell lines. A scratch wound assay was used to assess tumor cell migration while immunofluorescence for phosphorylated histone H3 or cleaved caspase 3 was used to determine tumor cell proliferation and apoptosis, respectively.

**Results:**

Sunitinib and Regorafenib, but not Imatinib, were capable of significantly inhibiting the migration of both murine and human luminal-like and claudin-low breast cancer cells while Masitinib inhibited migration in both human breast cancer cell lines. Sunitinib but not Regorafenib or Imatinib also significantly suppressed tumor cell proliferation in all four cell lines tested while Masitinib had no significant effect on human breast cancer cell proliferation. None of the PDGFR inhibitors consistently regulated mammary tumor cell apoptosis.

**Conclusion:**

Sunitinib, Regorafenib and Masitinib may prove clinically useful in inhibiting breast cancer cell migration and metastasis while only Sunitinib (and possibly Regorafenib in some breast cancer subtypes) is effective at inhibiting both migration and proliferation of breast cancer cells.

## Introduction

Platelet derived growth factors (PDGFs), as the name suggests, were originally purified from platelets [1–4]. PDGFs form homodimers consisting of A-, B-, C- and D-polypeptides or heterodimers consisting of A and B polypeptides. These ligands induce signaling by binding to one of two PDGFR receptor isoforms, PDGFRα and PDGFRβ. PDGFRα binds all members of the PDGF dimers other than D-polypeptides while PDGFRβ bind the B- and D-polypeptides [5]. PDGFRs are tyrosine kinase receptors and ligand binding induces receptor dimerization and intracellular signaling including activation of PLCγ, Src, SHP-2, RasGAP, PI3-kinase and STAT proteins [5]. These signaling pathways regulate cell proliferation, survival, chemotaxis and differentiation.

PDGFRs are typically not expressed in normal epithelial cells but are expressed in fibroblasts and smooth muscle cells [6] where these receptors regulate physiologic processes such as wound healing, inflammation and angiogenesis [7]. Tumors associated with enhanced PDGFR signaling include sarcomas, gastrointestinal stromal tumors and several types of leukemias [8].

With respect to breast cancer, less is known about the importance of PDGFs or PDGFRs. Studies have shown that PDGF-D is upregulated in invasive breast cancer while PDGF-BB is found at higher levels in patients with breast cancer compared to those with benign breast disease and the expression PDGFRs is a predictor of poor prognosis [9–11]. Additionally, more recent research has found that PDGF signaling is elevated in breast cancer cells that have become resistant to endocrine therapy [12,13]. Another potential role of PGDFR signaling in breast cancer is during epithelial to mesenchymal transistion (EMT). EMT is a process whereby epithelial cells acquire a more mesenchymal phenotype and gene expression profile and EMT has been implicated in more aggressive/metastatic breast cancers [14–17].

A number of agents capable of inhibiting PDGFR signaling have been developed including Sunitinib malate (Sutent), BAY 73–4506 (Regorafenib) and Imatinib mesylate (Gleevec). Sunitinib malate inhibits PDGFRβ, VEGFR2, Kit and FLT-3 [18–20]. This agent is currently FDA approved for the treatment of gastrointestinal stromal tumors, pancreatic neuroendocrine tumors and renal cell carcinoma and is currently in clinical trials for the treatment of breast cancer (www.cancer.gov). Regorafenib is FDA approved for the treatment of colorectal cancer and gastrointestinal stromal tumors (www.cancer.gov). Regorafenib can bind to and inhibit PDGFRβ as well as VEGFR2, VEGFR3, Ret, Kit and Raf [21,22]. Imatinib mesylate is FDA approved for the treatment of acute lymphoblastic leukemia, chronic eosinophilic leukemia, chronic myelogenous leukemia, germatofibrosarcoma protuberans, gastrointestinal stromal tumor, myelodysplastic/myeloproliferative disorders and systemic mastocytosis. Imatinib mesylate inhibits PDGFRα/β, c-kit and bcr-abl (www.cancer.gov). A more recently described inhibitor of PDGFRα and PDGFRβ is Masitinib. Masitinib inhibits Kit, PDGFRα, PDGFRβ and Lyn [23]. Masitinib is not FDA approved for any human cancers but is approved for use in canine mast cell tumors [24].

We have previously shown that PDGFRs were elevated in a murine mammary tumor cell line that has mesenchymal characteristics and closely resembles the human breast cancer subtype, claudin-low [25–27]. Claudin-low tumors are similar to basal-like breast cancer in that they are typically estrogen receptor, progesterone receptor and HER2 negative and express a number of mesenchymal genes. Claudin-low tumors are differentiated from basal-like tumors by the higher expression of genes involved in immune response, cell communication, extracellular matrix, cell migration and angiogenesis and their lower expression of claudins 3, 4, 7, and occludin [28,29]. We also showed previously that knockdown of PDGFRα, or knockdown of both PDGFRs significantly inhibited migration but did not inhibit proliferation or promote apoptosis in our claudin-low murine mammary tumor cell line [25].

In the current manuscript we extended our previous work to determine whether inhibitors of PDGFR could suppress migration and survival of murine and human luminal and claudin-low mammary tumor cells. We found that Sunitinib malate and Regorafenib significantly inhibited migration of all 4 cell lines tested. Sunitinib was also capable of suppressing proliferation in all 4 cell lines while Regorafenib suppressed proliferation only in the human claudin-low breast cancer cell line. Imatinib only significantly inhibited migration in the human luminal breast cancer cell line and had no significant effect on proliferation in any of the cell lines. Masitinib inhibited migration of both human breast cancer cell lines but had no effect on proliferation or apoptosis.

## Materials and methods

### Cells and culture conditions

RJ345 and RJ348 cells were generated in our lab and have previously been described [30]. RJ345 and RJ348 cells were cultured in DMEM containing 10% FBS, 1 mmol/L sodium pyruvate, 10 mmol/L HEPES, 4 mmol/L glutamine, 2 mmol/L hydrocortisone, 5 μg/mL estrogen, 5 μg/mL prolactin, 10 μg/mL epidermal growth factor, 10 μg/mL insulin and 1% antibiotic/antimycotic. MCF-7 cells were maintained in α-MEM media containing 10% FBS and 1% antibiotic/antimycotic while MDA-MB-231 were maintained in RPMI media containing 10% FBS and 1% antibiotic/antimycotic.

### PDGFR inhibitors

Regorafenib (Bay 73–4506), Imatinib mesylate (Gleevec), Masitinib (AB1010), and Sunitinib malate (Sutent) were purchased from Selleck Chemicals (Houston TX). All inhibitors were re-suspended in DMSO.

### Scratch wound assay

Cells were plated in 6-well plates in fully supplemented media such that they were 90-100% confluent at the time the scratch was performed. Once the scratch was made the media was removed and replaced with fully supplemented media containing different concentrations of Masitinib, Regorafenib, Sunitinib or Imatinib. Images of the scratch were captured immediately after the scratch was induced and 24 or 48 hrs after drug administration. Percentage of the wound closed was calculated using ImageJ software (NIH). All drugs were diluted such that each well contained 0.1% DMSO and the control well contained 0.1% DMSO.

### Immunofluorescence

Cells were plated in fully supplemented media on sterile glass coverslips in 6 well dishes at 20-30% confluence. Cells were allowed to adhere overnight and then the medium was removed and replaced with fully supplemented media containing Masitinib, Regorafenib, Sunitinib, or Imatinib. All drugs were diluted such that each well contained 0.1% DMSO and the control well contained 0.1% DMSO. Twenty-four hours later the media was removed, the cells washed 3 times in ice cold PBS and then fixed in 10% neutral buffered formalin for 1 hour at room temperature. Cells were washed 3 times in PBS and blocked with 5% BSA in PBS containing 0.1% Tween 20. For proliferation, cells were incubated with a 1:2000 dilution of an antibody against phospho-histone H3 (S10) (Abcam Inc, Toronto, ON; cat# ab14955) overnight at 4°C. For apoptosis, cells were incubated with a 1:500 dilution of an antibody against cleaved caspase 3 (EMD Millipore, Billerica, MA; cat# AB3623) overnight at 4°C. The cells were then washed twice in PBS and incubated with a 1:200 dilution of the appropriate fluorescently labeled secondary antibody for 1 hour at room temperature. Cells were washed, counterstained with DAPI and visualized using an Olympus BX61 fluorescent microscope (Olympus, Center Valley, PA) using Metamorph imaging software (Molecular Devices, Sunnyvale, CA). The number of positive cells and total number of cells were counted from at least 3 independent experiments.

### Statistics

An ANOVA followed by a Dunnett’s posthoc analysis was performed to determine statistically significant values. Values were considered significant when p < 0.05.

## Results

We have previously shown that RJ348 cells have characteristics similar to human claudin-low breast cancer while RJ345 cells resemble human luminal breast cancer [26]. We have also previously shown that RJ348 cells have elevated levels of PDGFRα and PDGFRβ compared to RJ345 cells and RNAi mediated knockdown of PDGFRs inhibited migration of RJ348 cells [25]. Although useful as a scientific tool, RNAi has limited therapeutic potential. Therefore, we evaluated the ability of several PDGFR inhibitors to regulate proliferation, apoptosis and migration in luminal-like and claudin-low breast cancer cells.

**Figure 1:**
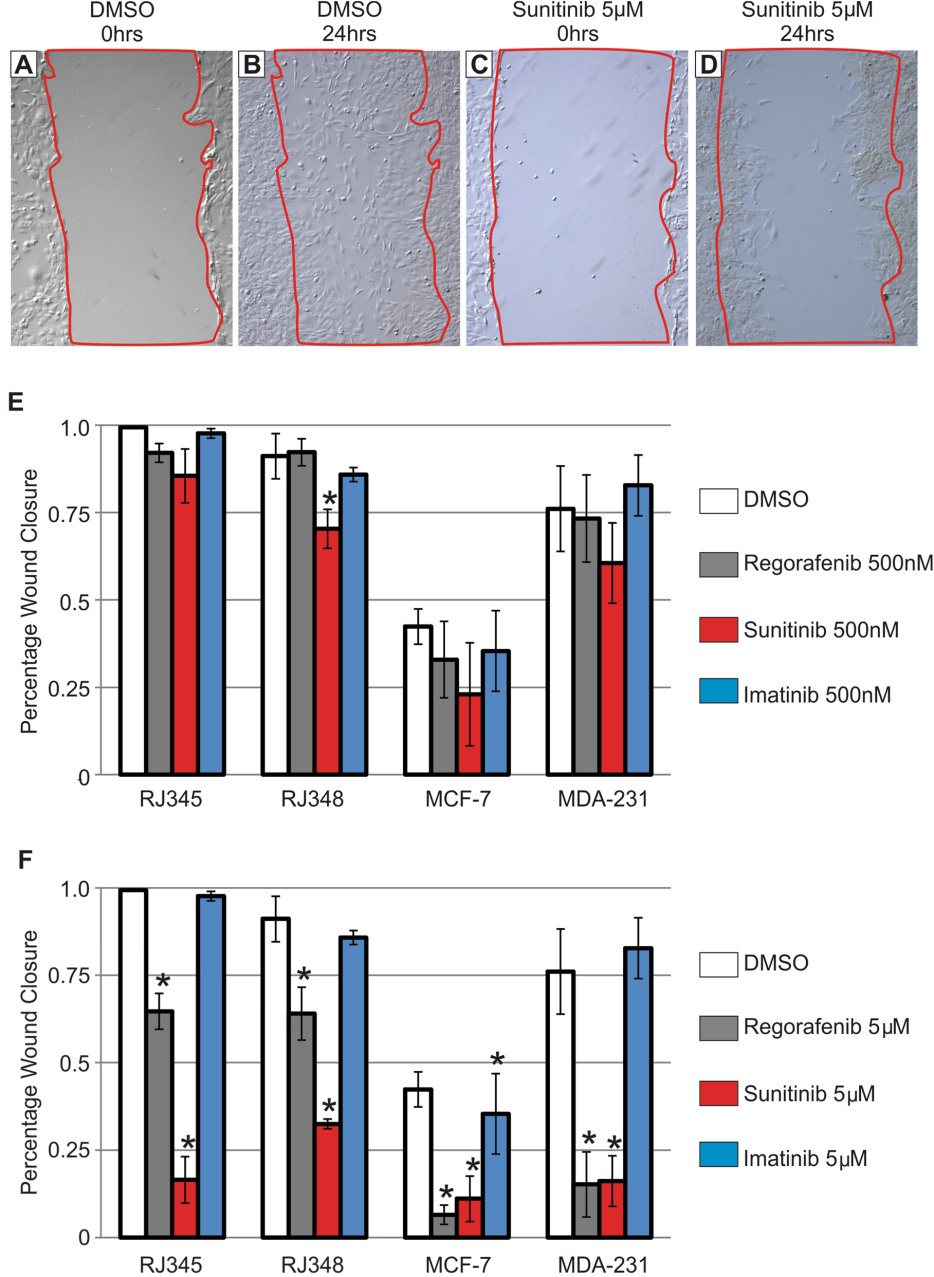
**Sunitinib and Regorafenib inhibit mammary tumor cell migration.** A scratch wound assay was performed on RJ345, RJ348, MCF-7 and MDA-MB-231 cells treated with 500 nM or 5 μM of Regorafenib, Sunitinib or Imatinib. **(A-D)** shows representative scratch wounds from RJ348 cells at time 0 **(A,C)** and 24 hrs **(B,D)** after the initiation of the scratch when the cells were treated with the vehicle control, DMSO **(A,B)**, or 5 μM of Sunitinib **(C,D)**. The red line indicates the initial scratch wound. The percentage of the wound closed was quantified from a minimum of 3 independent replicates and is expressed as mean ± SEM when treated with **(E)** 500 nM or **(F)** 5 μM of Regorafenib, Sunitinib or Imatinib. Wounds were created and inhibitors were added immediately. Wounds were evaluated at 24 hrs after inhibitor administration for the RJ348 cells and 48 hrs for RJ345, MCF-7 and MDA-MB-231 cells. *Indicates values that were significantly different (p < 0.05) from the DMSO control.

Since the most dramatic effect observed following PDGFR knockdown in the RJ348 cells was an inhibition of tumor cell migration [25] , tumor cell migration was evaluated using a scratch wound assay and Regorafenib, Sunitinib and Imatinib were tested at 100 nM, 500 nM and 5 μM. All of the inhibitors were re-suspended in DMSO and all dilutions were performed in DMSO such that each well received the inhibitor and 0.1% DMSO. Control wells contained 0.1% DMSO. Figure 1A-D shows representative scratch wounds for RJ348 cells at time 0 (when the scratch was made) and 24 hours after inducing the wound in cells treated with DMSO or Sunitinib at 5 uM. The bar graphs (Figure 1E,F) represent the percent of the wound that closed 24 hrs after wounding for the RJ348 cells and 48 hrs after wounding for the RJ345, MCF-7 and MDA-MB-231 cells. The murine claudin-low mammary tumor cell line, RJ348, migrated more rapidly than the luminal-like mammary tumor cell line, RJ345. In fact, the RJ348 cells migrated so quickly that the control cells (DMSO) completely closed the wounds by 48 hrs and thus the 24 hr time point was used for these cells. The human claudin-low cell line, MDA-MB-231, migrated more quickly than the luminal breast cancer cell line MCF-7 as illustrated by comparing the DMSO controls for the two cell lines. Regorafenib, Sunitinib and Imatinib failed to significantly reduce tumor cell migration when administered at 100 nM (data not shown). Only Sunitinib significantly reduced migration at 500 nM and this was only observed in the RJ348 cells (Figure 1E). At 5 μM, both Sunitinib and Regorafenib significantly inhibited migration in all cell lines while Imatinib only inhibited MCF-7 migration (Figure 1F).

**Figure 2:**
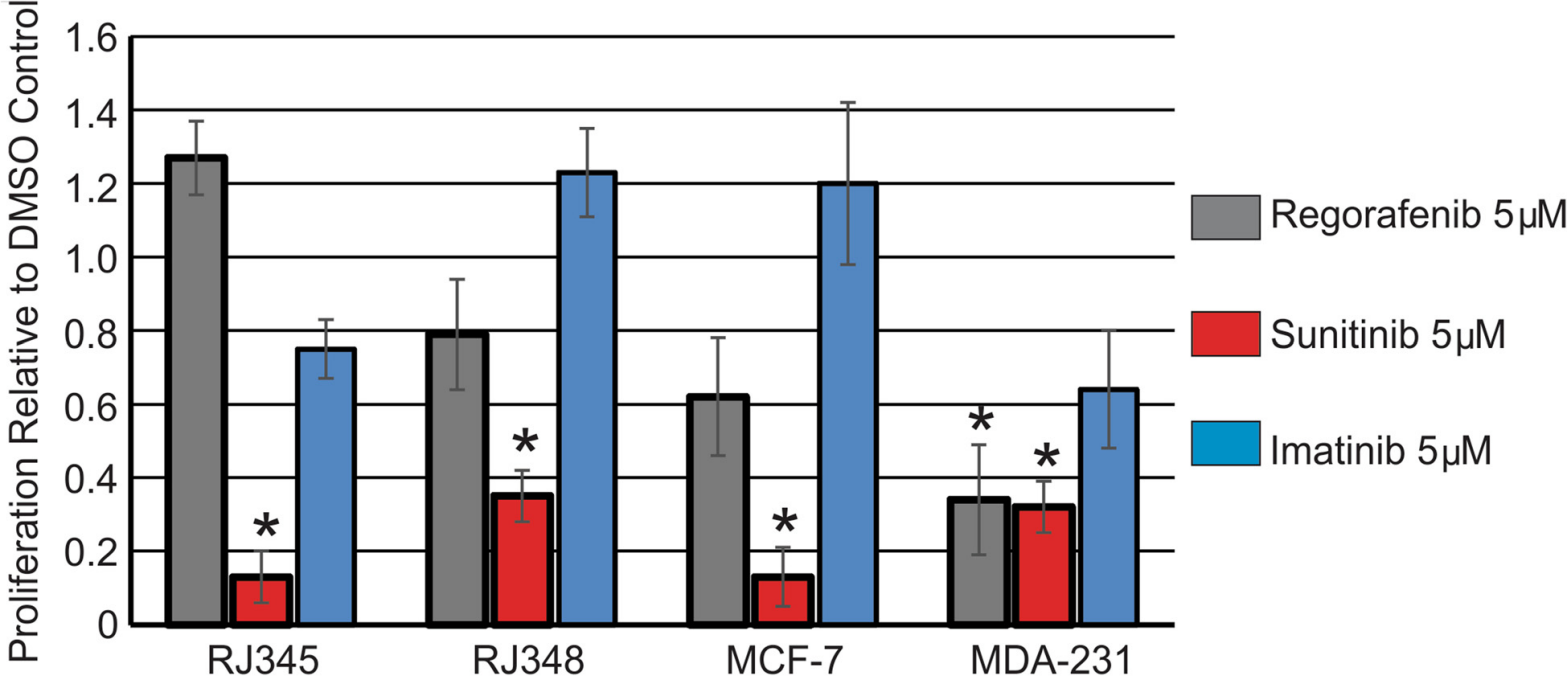
**Sunitinib inhibits mammary tumor cell proliferation.** Cell proliferation was assessed using phosphorylated histone H3 (S10) immunofluorescence. The bar graph represents the mean ± SEM of the percentage of positively stained cells relative to the DMSO control (n ≥ 3). *Indicates values that were significantly different (p < 0.05) from the DMSO control.

Next, cell proliferation in response to 5 μM of each inhibitor was evaluated. Figure 2 shows cell proliferation relative to the DMSO control and it was observed that 5 μM of Sunitinib significantly inhibited cell proliferation in all 4 cell lines while Regorafenib (5 μM) significantly inhibited proliferation only in MDA-MB-231 cells. Imatinib (5 μM) did not significantly inhibit proliferation in any of the cell lines.

**Figure 3:**
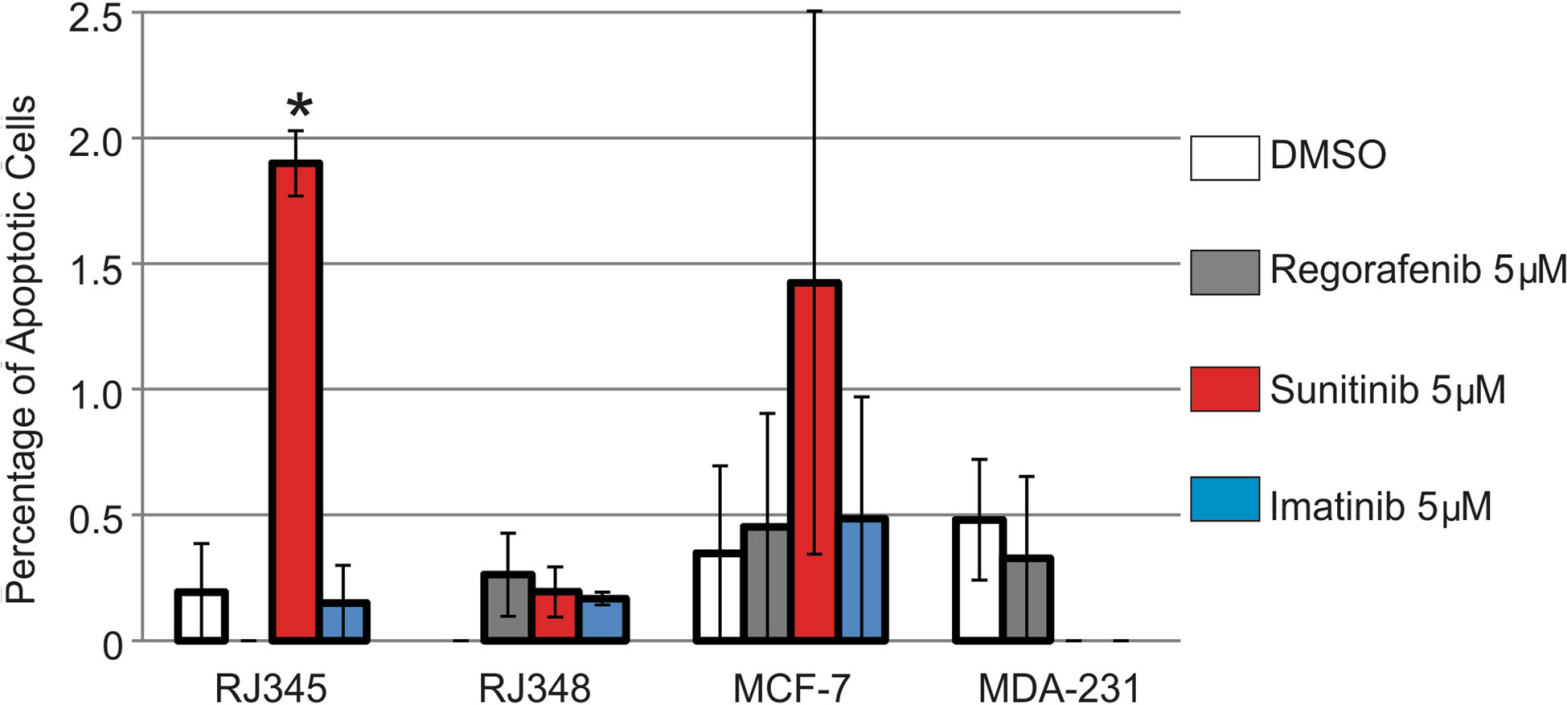
**Sunitinib promotes apoptosis in RJ345 cells.** Cell apoptosis was assessed using cleaved caspase 3 immunofluorescence. The bar graph represents the mean ± SEM of the percentage of positively stained cells (n ≥ 3). *Indicates values that were significantly different (p < 0.05) from the DMSO control.

When apoptosis was evaluated, only 5 μM of Sunitinib significantly induced apoptosis and this was only observed in the RJ345 cells. The graph in Figure 3 represent the percentage of apoptotic cells in each treatment and as can be seen in the graph, the levels of apoptosis were very low. The RJ345 cells treated with 5 uM of Sunitinib had the highest level of apoptosis and even this was only observed in approximately 1.8% of the cells.

**Figure 4:**
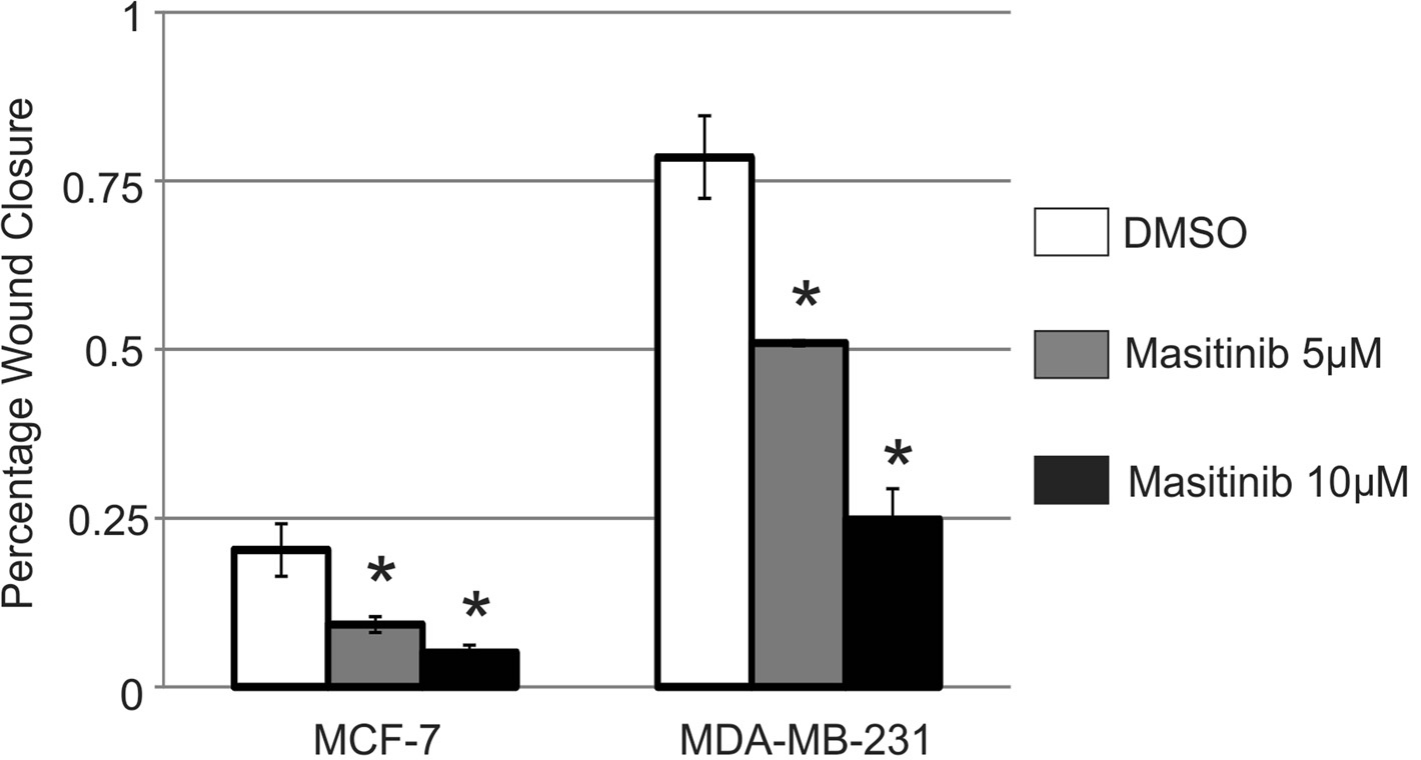
**Masitinib inhibits migration of MCF-7 and MDA-MB-231 cells.** The bar graph represents the percentage of the wound closed from 3 independent scratch wound assays and is expressed as mean ± SEM when treated with (grey bar) 5 μM or (black bar) 10 μM of Masitinib. Masitinib was added to the cells immediately after the creation of the wound and wounds were evaluated at 48 hrs after inhibitor administration. *Indicates values that were significantly different (p < 0.05) from the DMSO control.

A more recently identified inhibitor of PDGFRα and PDGFRβ, Masitinib, was evaluated at 5 μM and 10 μM in MCF-7 and MDA-MB-231 cells and was found to significantly inhibit migration in both MCF-7 and MDA-MB-231 cells at both 5 μM and 10 μM (Figure 4) but did not significantly impact proliferation or apoptosis in either cell line at either concentration (data not shown). Masitinib was not evaluated in the murine mammary tumor cell lines.

## Discussion

Our previous study showed that murine claudin-low mammary tumor cells expressed elevated levels of PDGFRα and PDGFRβ compared to murine luminal mammary tumor cells. In addition, knockdown of PDGFRα or PDGFRβ significantly reduced migration but not proliferation of the claudin-low mammary tumor cells [25]. Although targeting PDGFRs with RNAi is a useful scientific tool, this approach is unlikely to be clinically viable. Therefore we examined the efficacy of several PDGFR small molecule inhibitors in both our murine mammary tumor cell lines and in human breast cancer cell lines. The cell lines were chosen such that there would be one murine luminal-like (RJ345) and one human luminal cell line (MCF-7) as well as one murine (RJ348) and one human (MDA-MB-231) claudin-low cell line. Since a purely selective PDGFR inhibitor does not currently exist, we selected 3 inhibitors that target PDGFR and are currently used clinically. These inhibitors were Regorafenib (Bay 73–4506), Imatinib mesylate (Gleevec) and Sunitinib malate (Sutent). In addition, Masitinib, which is not clinically used for human cancers but is used for canine mast cell tumors [24] was evaluated in the human breast cancer cell lines. Masitinib was included in the study as it inhibits PDGFRα and PDGFRβ (similar to Imatinib) while Regorafenib and Sunitinib only inhibit PDGFRβ.

There were a number of surprising findings in this study. The first surprising finding was that Regorafenib and Sunitinib were equally capable of suppressing mammary tumor cell migration and proliferation in both luminal-like and claudin-low cell types. Based on western blots from our previous study [25] we anticipated either (i) the claudin-low tumor cells would be more sensitive to PDGFR inhibition as these cells express higher levels of PDGFR and thus were more dependent on PDGFR signaling for migration and proliferation or (ii) the higher levels of PDFGR found in the claudin-low tumor cells would render these cells more resistant to PDGFR inhibition as higher concentrations of Sunitinib or Regorafenib would be required to competitively block PDGFR signaling. The percentage of migration inhibited was similar in the luminal-like cell lines compared to their claudin-low counterparts indicating that PDFGR expression did not predict sensitivity to PDGFR inhibition. However, it does appear that both luminal-like and claudin low breast cancer cells rely on PDGFR signaling for migration and that PDGFR inhibitors can effectively suppress migration even in cells that express high levels of PDGFRs.

The second surprising finding was that Sunitinib inhibited proliferation of the claudin-low cell lines. At least for the RJ348 cells, our previous study showed that knockdown of PDGFR either had no effect on proliferation or induced a slight increase in proliferation. In the current study Sunitinib inhibited proliferation of all four cell lines. This effect is likely results from inhibition of kinases other than PDGFRβ.

The third surprising finding was that Imatinib was less effective at inhibiting migration than either Sunitinib or Regorafenib. Our previous study showed that concomitant knockdown of both PDGFRα and PDGFRβ was the most effective strategy for inhibiting RJ348 migration and knockdown of PDGFRα was more effective at suppressing migration than PDGFRβ knockdown [25]. Since Imatinib targets both PDGFRα and PDGFRβ while Sunitinib and Regorafenib only target PGDFRβ, we anticipated that Imatinib would be the most effective inhibitor at suppressing tumor cell migration. The lack of effect of Imatinib could be due to the higher IC_50_ for PDGFR inhibition (100 nM) compared to Sunitinib (PDGFRβ IC_50_ of 2 nM) and Regoragenib (PDGFRβ IC_50_ of 22 nM). It is also possible that a kinase targeted by both Sunitinib and Regorafenib, but not Imatinib, is critical for migration and proliferation in the tumor cells tested. However, the fact that Masitinib, which inhibits both PDGFRα and PDGFRβ as well as c-kit (similar to Imatinib), significantly inhibited migration of both human breast cancer cell lines, but had no effect on proliferation or apoptosis argues against the IC_50_ or other kinase targets limiting Imatinib’s efficacy. Masitinib’s IC_50_ for PDGFRα and PDGFRβ is reported to be higher than Imatinib’s and the kinases inhibited by Masitinib are also inhibited by Imatinib. Therefore, it appears that inhibition of PDGFRβ can suppress breast cancer cell migration but does not regulate breast cancer cell proliferation. This would be consistent with our observations using PDGFR RNAi in RJ345 and RJ348 cells in that inhibition of PDGFRs can suppress migration but not proliferation [25]. The ability of Sunitinib and Regorafenib to inhibit breast cancer cell proliferation presumably results from inhibition of a kinase other than PDGFRβ. The only kinase reported to be targeted by both Sunitinib and Regorafenib (that is not targeted by Masitinib) is VEGFR2 and thus this receptor deserves further investigation as an anti-proliferative agent in the treatment of breast cancer.

A review of the literature revealed that Regorafenib has not been tested on MCF-7 or MDA-MB-231 cells in vitro. One manuscript did evaluated the effects of Regorafenib on MDA-MB-231 cells in vivo and found that Regorafenib at 3 mg/kg and higher significantly inhibited MDA-MB-231 xenograft growth [21]. For Sunitinib, one manuscript examined MCF-7 and MDA-MB-231 proliferation in vitro. This study showed that 5 uM of Sunitinib significantly inhibited proliferation of both MCF-7 and MDA-MB-231 cells as assessed using a H^3^-thymidine assay. Migration was not evaluated [31]. Three studies examined the effects of Imatinib on MCF-7 and MDA-MB-231 cells in vitro. One study showed that the IC_50_ of Imatinib in both MCF-7 and MDA-MB-231 cells was approximately 7 uM [32]. At 5 μM, Imatinib reduced MDA-MB-231 proliferation to approximately 75% of control and MCF-7 proliferation to approximately 85% of control [32]. In the second study it was found that imatinib at concentrations of 3 uM and higher could inhibit proliferation of both MCF-7 and MDA-MB-231, however, these studies were performed in serum-free conditions [33]. The final study showed that Imatinib could inhibit MCF-7 but not MDA-MB-231 cell migration at 48 hrs after administration of 6 uM of Imatinib when the cells were cultured in 1% serum [34]. Therefore, our findings on Regorafenib, Sunitinib and Imatinib are consistent with the limited number of breast cancer studies using these agents. No study has evaluated Masitinib treatment on MCF-7 or MDA-MB-231 cells. Therefore, this is the first study to demonstrate that Masitinib can inhibit migration of luminal and claudin-low human breast cancer cells but has no significant impact on proliferation or apoptosis.

Pharmacokinetic studies in mice and rats have shown maximal plasma concentrations of Sunitinib that can be achieved are in the range of 3–3.5 μM [35]. In humans, administration of more than 50 mg of Sunitinib daily is associated with significant toxicity and at 50 mg/day, plasma levels of Sunitinib range from 50–90 ng/ml (~0.1 μM-0.17 μM) [36]. However, a study by Gotink et al. [37] has shown that intratumoral concentrations of Sunitinib are more than 10-fold higher than plasma samples. In mice administered Sunitinib at 40 mg/kg/day, plasma concentrations were approximately 1 μM while tumor concentrations were approximately 11 μM. Similarly in humans administered 37.5-50 mg of Sunitinib per day, plasma concentrations were approximately 0.3 μM while tumor concentrations were approximately 9.5 μM. For Regorafenib and Imatinib, plasma concentrations of 2522 ng/ml (5.2 μM) and 3000 ng/ml (5.1 μM) respectively, have been reported [38,39]. Plasma concentrations for Masitninb in humans has not been reported but concentrations of 1886 ng/ml (3.9 μM) have been reported in cats [40]. Therefore, the concentrations used in this study are within the range of those clinically achievable.

Although clinical trials using Sunitinib in the treatment of breast cancer have been disappointing thus far [41–43] a recent study from the Weinberg lab reinforces the importance of PDGFR signaling in breast cancer. These investigators found that that EMT and the enrichment of breast cancer stem cells was associated with a switch from EGFR signaling to PDGFR signaling [44]. In addition this study showed that PDGFR expression was elevated in mammary tumor cells with stem cell properties and PDGFR inhibitors could selectively target breast cancer cells with stem cell properties [44]. Therefore, additional research is required to identify specific PDGFR inhibitors and how best to utilize these agents for targeting different breast cancer subtypes or specific cell types (ie stem cells) in breast cancers.
